# A Review of Data Quality Assessment in Emergency Medical Services

**DOI:** 10.2174/1874431101812010019

**Published:** 2018-05-31

**Authors:** Mehrnaz Mashoufi, Haleh Ayatollahi, Davoud Khorasani-Zavareh

**Affiliations:** 1 PhD Student of Health Information Management, School of Health Management and Information Sciences, Tehran Iran University of Medical Sciences, Tehran, Iran; 2 School of Health Management and Information Sciences, Iran University of Medical Sciences, Tehran, Iran; 3 Safety Promotion and Injury Prevention Research Center, Shahid Beheshti University of Medical Sciences, Tehran, Iran; 4 Department of Health in Disaster and Emergency, School of HSE, Shahid Beheshti University of Medical Sciences, Tehran, Iran

**Keywords:** Data accuracy, Assessment, Emergency Medical Services, Medical Records, Health care, Hospital records

## Abstract

**Introduction:**

Data quality is an important issue in emergency medicine. The unique characteristics of emergency care services, such as high turn-over and the speed of work may increase the possibility of making errors in the related settings. Therefore, regular data quality assessment is necessary to avoid the consequences of low quality data. This study aimed to identify the main dimensions of data quality which had been assessed, the assessment approaches, and generally, the status of data quality in the emergency medical services.

**Methods:**

The review was conducted in 2016. Related articles were identified by searching databases, including Scopus, Science Direct, PubMed and Web of Science. All of the review and research papers related to data quality assessment in the emergency care services and published between 2000 and 2015 (n=34) were included in the study.

**Results:**

The findings showed that the five dimensions of data quality; namely, data completeness, accuracy, consistency, accessibility, and timeliness had been investigated in the field of emergency medical services. Regarding the assessment methods, quantitative research methods were used more than the qualitative or the mixed methods. Overall, the results of these studies showed that data completeness and data accuracy requires more attention to be improved.

**Conclusion:**

In the future studies, choosing a clear and a consistent definition of data quality is required. Moreover, the use of qualitative research methods or the mixed methods is suggested, as data users’ perspectives can provide a broader picture of the reasons for poor quality data.

## 1 INTRODUCTION

The use of data is the basis for conducting a variety of activities and making decisions on operational and strategic levels in different organizations [1]. While high quality data is essential for the success of organizations, the drawbacks of low quality data are increasingly experienced by different companies and institutions [2] and have great impacts on their efficiency and effectiveness [3].

Generally, data quality refers to the extent that the data fulfill users’ expectations and suit its intended purposes [4]. Data quality is also an important issue in the field of healthcare and healthcare information technology [HIT] [5]. In fact, the quality assurance of the data in healthcare systems is an emphasis on the continuity of the quality of care [6], and the technologies that support clinical care need accurate and complete data [7].

Among different medical specialty, the quality of data is of critical importance in the field of emergency medicine. The unique characteristics of emergency care services, such as high turn-over, repetitive assignment of care from one provider to another, high fluctuation in the number of patients, and the attendance of unfamiliar inpatients in the emergency departments may increase the possibility of making errors in this setting [8, 9]. As a result, emergency medicine is regarded as a challenging field of healthcare services, and the generation of high-quality data is one of the main concerns for performance measurement in this setting [10].

The practice of physicians in the emergency departments is accompanied with multiple pauses that are related to conducting various duties and simultaneous communication between healthcare providers [11]. From the emergency medicine perspective, a pause in the process is an indicator for the potential error in the cycle of data documentation. Therefore, the regular assessment of data quality is crucial to avoid the adverse effects of using low quality data in the process of decision making [12, 13]. Moreover, according to Obermeyer ***et al***., compared to high-income countries, in which clinical and emergency care have dramatically improved during the last decades, in developing countries there is still a lack of data from the field of emergency medicine that has made it difficult to make new investments in emergency care. Hence, any improvement to emergency care in these countries will require advances in data collection methods and data quality. Tanzania and South Africa are among the countries which have started working on emergency data quality [14].

To assess data quality, it is pivotal to have a common grasp of its concept. Several investigators have pointed out a lack of a common definition for the data quality in healthcare. However, data quality is defined frequently as fitness for use, *i.e.* the extent the data can be used for its user purposes [15]. It is also suggested that data is suitable for application when it can provide he required information for its users [16, 17].

Data quality assessment has a great impact on data quality development. During the assessment process, the main reasons for data deficiency and errors and the necessary control processes will be identified which eventually may lead to improve the quality of data [18]. Since data quality is a multidimensional concept, it is important to pay more attention to the methods of data quality assessment [19, 20]. According to the literature data quality assessment can be performed using quantitative and qualitative methods [4, 21]. Objective and subjective assessment methods are other approaches to assess the quality of data. The objective approach focuses on the evaluation of the stored data by calculating specific quality criteria [21, 22], the subjective approach, reflects the requirements of the beneficiaries [data collectors, administrators, and data users] and their understanding and expectations of using the data. This approach is a supportive method for the objective assessment of data quality and investigates data quality and its dimensions in a real world setting [23].

In 2014, Chen ***et al***. conducted a systematic review of data quality assessment methods in public health information systems. The researchers noted that public health information systems whether paper or computer-based, are the repositories of public health data and their data are frequently used to monitor public health outcomes [21]. However, as noted before, the context of emergency medical services is different and the quality of data is pivotal at the point of care. Even a small delay in retrieving health care information makes that information essentially useless for emergency care [24]. While there are a wide variety of electronic systems with different functionalities available for use in emergency medical services, these variations may affect physicians' decision-making, clinicians' workflow, communication, and the overall quality of care and patient safety [8]. Moreover, the study conducted by Chen **et al**. was general and the concept of data quality in the context of emergency medical services was not discovered in particular [21]. In the current study, the researchers aimed to identify the assessment approaches, the main dimensions of data quality and the status of data quality in the emergency medical services.

## 2 METHODS

This review was conducted in 2016. Related articles were identified by searching databases, such as Scopus, Science Direct, PubMed and Web of Science using Boolean operators (AND/OR) with the following entry terms: quality, data accuracy, patient records, medical records, assessment, evaluation, health information, health information management, emergency medical services, emergency care, emergency medicine, and pre-hospital emergency care. An example of the search strategy in PubMed was as follows:

(“quality”[Title/Abstract] OR “data accuracy”[Title/Abstract] OR “patient records”[title/Abstract] OR “medical records”[Title/Abstract] OR “health information”[title/abstract] OR “health information management”[Title/Abstract]) AND (“assessment”[Title/Abstract] OR “evaluation”[Title/Abstract]) AND (“emergency medical services”[Title/Abstract] OR “emergency care”[Title/Abstract] OR “emergency medicine”[Title/Abstract] OR “pre-hospital emergency medicine”[Title/Abstract])

**Fig. (1):**
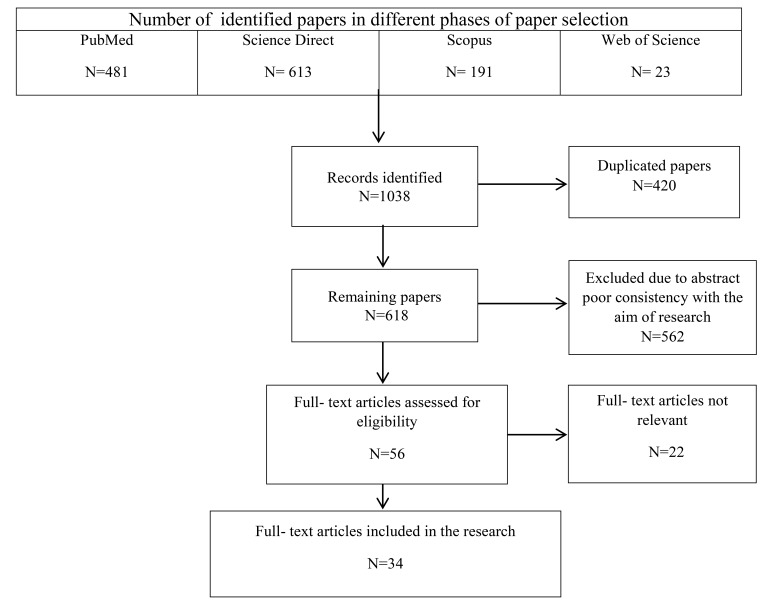
The process of selecting papers for the research.

All of the review and research papers related to data quality assessment in the emergency medical services published between 2000 and 2015 were included in the study. The non-English papers, books and editorial letters were excluded. Initially, 1038 papers were retrieved of which 420 duplicated papers were removed by using EndNote X7 software. The remaining 618 papers were screened based on titles, abstracts, and the relevance to the study subject. At this stage, 56 relevant full text papers were identified. Having read the papers, finally, 34 articles were selected to be reviewed (Fig. **1**). Two researchers (MM) and (HA) involved in the process of paper selection and the results were discussed with the third researcher (DKZ). For each paper, information about the author, publication year, study objectives, data quality dimensions, methodology, and a summary of findings were extracted.

## 3 RESULTS

Among 34 selected papers, 32 articles were published in the journals and two articles were presented in conferences. Nineteen articles were published between 2010 and 2015. Published papers were from the following countries: USA (21 papers), Australia (eight papers), England (two papers), Norway (one paper), Saudi Arabia (one paper), Canada (one paper). In the following sections, more details about the selected papers are presented.

### 3.1 Research Objectives

The primary objective of all articles was to assess the quality of data; however, some of them set some secondary objectives, too. For instance, some papers investigated the quality of data and its impact on preparing guidelines to support clinical [25] and managerial judgements [26], the concordance of trauma registry and hospital records [27], functional parameters in the emergency care services [28], and designing a dashboard for the emergency department [29] (Table **1**). In four studies, data quality assessment was conducted based on the work processes and the flow of information [29-31] and some others examined the effect of interventions on the quality of data. Three studies were performed to introduce new techniques for assessing the quality of data. These techniques included simulation [phenomenology] [28], and narrative texts analysis [32]. A number of papers assess the quality of data to determine information gaps. In these papers, completeness, accessibility of information, and documentation errors were the main areas to be assessed [33-39].

In two articles, a data quality assessment model was presented and discussed. According to this model, data quality assessment can be conducted with respect to three dimensions which are technical, organizational and individual [29, 31]. Although most papers had assessed the quality of data in the emergency departments, a few papers had focused on the quality of data in pre-hospital emergency care services [33, 40-43].

### 3.2 Data Quality Dimensions

Having reviewed the literature it was revealed that among different dimensions of data quality, five dimensions have received more attention in healthcare. These dimensions were accuracy, timeliness, completeness, relevancy, and consistency [44]. The definitions of these dimensions are presented in Table **2**.

In the current study, the findings showed that in the emergency care services, the main dimensions of data quality were completeness, accuracy, consistency, accessibility, and timeliness. According to Table **3**, all papers had investigated one or more dimensions of data quality.

Interestingly, the results showed that the definitions of data quality dimensions were occasionally different in various studies. For instance, terms like missing, incompleteness, information gap, and omission were used to address the completeness dimension in some studies and invalid values were regarded as missing data (incompleteness) in one study [45] Similarly, the lack of common understanding among researchers and differences in definitions could be seen in other data quality dimensions, such as accuracy, consistency and accessibility.

### 3.3 Methods of Data Quality Assessment

The objective or quantitative methods were the most common method used to assess the quality of data. Among quantitative methods (*e.g.*, retrospective, cross-sectional survey), retrospective and cross-sectional studies with statistical data were the most common ones. The subjective or qualitative methods (including review of publications and documentations, interviews with key informants, and field observations) were used only in four papers [12, 29, 31, 58], and the mixed methods [quantitative and qualitative] were applied in three studies to assess the quality of emergency medical services data [37, 46, 47]. In these articles, the quantitative methods had been used to measure the dimensions, and the qualitative methods had been utilized to investigate users’ perspectives about the quality of data or the main reasons for the information gap.

### 3.4 Reported Findings After Data Quality Assessment

The results of the studies could be categorized in three categories. The first category was related to assessing different dimensions of data quality and the following findings were reported by the researchers: completeness (between 30% and 100%), accuracy (between 57% and 99%), consistency (between 54% and 98%), accessibility (between 50% and 98.7%) and data errors were found in timestamps [28, 48, 49].

The second category was related to the studies in which an intervention was performed to improve data quality. In this category, different approaches, such as simulation (phenomenology) [28] and data linkage [40] were used to improve data quality. Some researchers examined the effect of interventions on the quality of data. The interventions included the use of a structured encounter form for documentation [50], the use of a standardized patient transfer form [35, 51], a patient-centred health information technology (a mobile kiosk) [7], the use of Electronic Health Records (EHR) [48], the use of vital signs data recorder [VSDR] technology in pre-hospital care [52], electronic documentation [53], and narrative texts analysis [32]. One study applied educational interventions to develop documentations [34] and one study examined the role of pharmacists in identifying discrepancies in medication histories [54].

The third category was related to the papers which focused on the data quality issues in the emergency medical services. These issues included information gaps and related causes, data quality and dashboard development projects, data quality issues emerged during data collection and reporting processes, data quality issues from the emergency department staff perspectives.

## 4 DISCUSSION

Emergency medicine is an information intensive speciality in which timely access to accurate patient information is of high importance [55]. Therefore, data quality assessment is necessary in this field. In the current study, papers related to data quality assessment in the field of emergency medical services were reviewed and the results showed that there were different and sometimes ambiguous definitions for data quality dimensions and related characteristics. The results are in line with the findings of other studies conducted by Chen **et al**. [21] and Michnik and Lo [56]. The lack of common understanding among researchers and differences in definitions of data quality dimensions have led to conducting various studies and obtaining different results. For example, in some papers, data availability was regarded as equivalent to completeness [25, 45, 49], accessibility [57], and missing data [58]. In one study, conflict was considered equivalent to inconsistency [58], while in another study it was regarded as data omission [54]. It seems that the definitions and characteristics of the data were different based on the intuition, past experiences, and assessment purposes [21]. Therefore, the use of ontology-based or standard definitions in the future studies can help to be able to compare the research methods and the results [21].

**Fig. (2):**
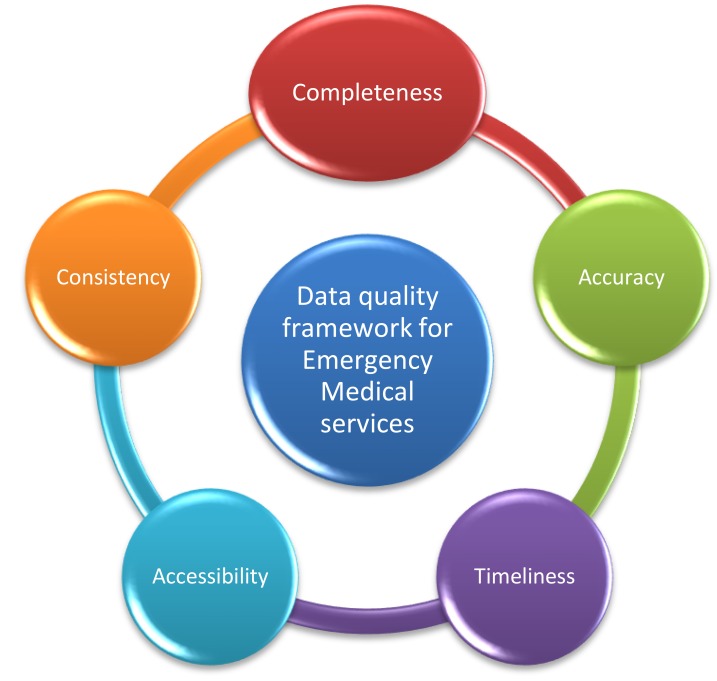
Data quality framework for emergency medical services.

According to the findings, the most common data quality dimensions assessed in the emergency medical services were completeness, accuracy, and consistency followed by accessibility and timeliness. In EMS, data completeness and data accuracy are important for improving the quality of care and for making right decisions. Similarly, data consistency is important to show the agreement between two or more sources of information and the possibility of data linkage between the primary and secondary care systems [59]. The linkage between data sources, in turn, can help to promote the quality of communication and information flow across the healthcare organizations [41]. The characteristics of emergency medical services also necessitate information being accessible for treating patients in a timely manner [55]. It seems that these data quality dimensions can be considered as a data quality framework for emergency medical services (Fig. **2**). According to Almutiry **et al**., the existing data quality frameworks are based on literature review, industrial experiences or intuitive understanding and the definition of a dimension may vary from one framework to another [6]. Moreover, the frameworks are too generic to adopt, may not reflect the nature of a domain, or may have some irrelative attributes. Therefore, such a data quality framework for emergency medical services can help to cover the main quality dimensions which are more relevant to the nature of emergency medical services. The proposed data quality framework is more comprehensive than the framework suggested by Chen **et al**. in which the main data quality dimensions were completeness, accuracy and timeliness [21].

Regarding the assessment methods, the findings demonstrated that there was no unique method to assess the quality of emergency medical services data and a variety of research methods was used by the researchers. This is consistent with the findings reported by Chen **et al**. [21]. However, the findings of the current study showed that the use of quantitative research methods had the highest frequency. In these studies, quantitative methods were mainly used to assess data accuracy, data consistency, data completeness, and data availability which are in line with the reported findings in other studies [53]. According to the literature, in order to assess different aspects of data quality, objective and subjective methods can be used. However, quality is a very subjective concept and depends on many other factors. As Pipino **et al**. noted assessing data quality requires the awareness of the fundamental principles and should be assessed by using both subjective and objective data quality metrics [18]. The use of mixed methods [quantitative and qualitative] and different information resources, such as files, organizational documents, and users' perspectives has also been suggested in other studies [19, 21, 60].

In fact, performing subjective and objective data quality assessments helps to compare the results of the assessments, to identify discrepancies, and to determine root causes of errors for determining and taking necessary actions for improvement [61]. In fact, the subjective assessment is a supplement to the objective assessment and is useful for designing effective strategies for improving data quality [15, 21, 22, 62].

The results also revealed that the quality of data could be affected by a number of technical, organizational, and individual factors [31, 48, 53]. Since, these factors may also influence data accessibility, they should be taken into account when designing information systems [55]. Overall, different studies showed that technical factors, such as ED information system user-interface and data extracting methods may influence the timiliness, accuracy, and completeness of data, while data consistency and data integrity are mainly affected by the organizational factors (*e.g.*, waiting time) and individual factors (*e.g.*, capturing all required data from patients] [29, 31]. The results showed to improve the quality of data in emergency medical services, different interventions can be made; however, most studies have used different types of health information technologies and structured forms. This shows that technical factors and information systems can be replaced with the traditional paper-based records; however, they need to be designed and implemented carefully as the quality of data can be easily affected by the quality of these system [55].

Overall, it can be concluded that data quality in emergency medical services deserves more attention, scientific research and investment. The data quality framework propsed in the current study can be used in different settings of emergnecy medical services to compare the quality of data and to understand how to improve the current situations. Moreover, it is a simple and clear framework which can be easily used by the health care practitioners in the field to examine the quality of data in their own settings. The results of this type of research can also help to develop better quality assurance strategies, to support health care delivery, and to improve patient care.

## CONCLUSION

Emergency medical services require high quality data to support healthcare delivery and decision making and data quality assessment is of great importance in this field. The results of the current study showed that due to the diversity of definitions and terminology used to assess the dimensions, characteristics, and attributes of data quality, comparing findings reported in different studies was difficult. Therefore, in the future studies, more attention should be paid to choose a clear and a consistent definition of data quality. In this study, a data quality framework was proposed to be used in the context of emergency medical services. The main data quality dimensions were completeness, accuracy, consistency, accessibility and timeliness. This framework shows that data consistency and data accessibility are two important quality dimensions for emergency medical services in addition to data completeness, accuracy and timeliness. Regarding the data quality assessment methods, the use of qualitative research methods or the mixed methods is suggested, because data users’ perspectives can provide a broader picture of data quality and related issues. Finally, emergency data quality needs to be assessed at different organizational levels using different resources. This approach can help to identify quality issues and the most appropriate interventions to improve data quality.

## Figures and Tables

**Table 1:** Summary of the reviewed papers.

**Authors ** **Year**	**Country**	**Objective**	**Quality Attributes **	**Method**	**Results**
Aronsky and Haug (2000)	America	To examine whether clinical data routinely available in a computerized patient record (CPR) can be used to drive a complex guideline that supports physicians in real time and at the point of care in assessing the risk of mortality for patients with community acquired pneumonia	Availability, Concordance	Quantitative/ Cross-sectional study	From a clinical perspective, the current level of data quality in the HELP System and its CPR supports the automation and the prospective evaluation of the Pneumonia Severity Index as a computerized decision support tool.
Stiell **et al*. * (2003)	America	To measure the prevalence of physician-reported information gaps for patients presenting to an emergency department	Completeness	Quantitative/ Cross-sectional study	Information gaps were present in almost one-third of the visits to emergency department. They were more common in sicker patients and were independently associated with a prolonged stay in the emergency department.
Jones **et al*. *(2003)	England	To determine whether narrative information in emergency department surveillance systems can be systematically interrogated to improve our understanding of the causes of injury	Accuracy completeness	Quantitative/ Cross-sectional study	The proportion of records carrying an informative emergency department code was higher in records containing narrative information.
Smith **et al*. * ** **(2004)	Australia	(i)To design and implement a quality assessment tool to determine the quality of the ambulance patient care record (PCR) information. (ii) To identify critical demographic and clinical items on the ambulance PCR that needed improvement	Completeness	Quantitative/ Retrospective cohort study	A quality assessment tool and associated user guide was developed .Three critical patient care record (PCR) components required improvement (patient details, observations and management).
Downing **et al*. *(2005)	England	To link ambulance services and Emergency Department (ED) data for assault patients, to look at the potential advantages of this linkage, and to investigate the quality of coding in the two data sets	Concordance	Quantitative/ Cross-sectional study	Data linkage between ambulance services and ED can increase the amount of information available in both data set.
Kanegaye e*t al.* (2005)	America	To measure the effect of introduction of a structured encounter form on the completeness of documentation of pediatric wound management in a teaching hospital	Completeness	Quantitative/ Prospective (Pre-post study)	The use of a structured complaint-specific form improved overall completeness of wound-care documentation (80% vs 68% for free text).
Gorelick **et al*. *(2005)	America	To determine the availability and completeness of selected data elements from administrative and clinical sources for emergency department (ED) visits in a national pediatric research network.	Availability	Quantitative/ Retrospective study	Data elements important in emergency medical care for children are frequently missing in existing administrative and medical record sources.
Nagurney **et al*. *(2005)	America	To describe and test a model that compares the accuracy of data gathered prospectively versus retrospectively among adult emergency department patients admitted with chest pain.	Completeness, accuracy	Quantitative/ Prospective and retrospective study	Information obtained retrospectively from the abstraction of medical records is less accurate than information obtained prospectively from patients. This study indicates that clinicians document elements of care delivered to patients very poorly.
McKenzie **et al*. * (2005)	Australia	To examine the concordance of trauma registry and hospital records in Queensland in 1998	Concordance Completeness, accuracy	Quantitative Retrospective	This study identified four main types of error including failure to identify relevant patients, inappropriate inclusion of patients, insufficient/inaccurate data in hospital records, insufficient/ inaccurate data in the trauma registry.
Considine **et al*. *(2006)	Australia	To examine the effect of written ED nursing practice standards augmented by an in-service education programme on the documentation of the initial nursing assessment	Completeness	Quantitative/ (Pre-post test study)	Written ED nursing practice standards improved emergency nurses’ documentation of the initial nursing assessment except oxygen saturation, heart rate or blood pressure.
Travers **et al*. *(2006)	America	To measure the time of availability of participating EDs’ diagnosis data in a state-based syndromic surveillance system.	Availability, Timeliness	Quantitative/ Prospective study	A majority of the ED visits transmitted to the state surveillance system did not have a diagnosis until more than a week after the visit. Reasons for the lack of timely transmission of diagnoses included coding problems, logistical issues and the lack of IT personnel at smaller hospitals.
Porter **et al*. *(2006)	America	(i)To identify the extent to which information provided by parents in the pediatric emergency department (ED) can drive the assessment and categorization of data on allergies to medications.(ii)To identify errors related to the capture and documentation of allergy data at specific process level steps during ED care.	Accuracy	Quantitative/ Observational study	There are significant gaps in the quality of information management regarding medication allergies in the pediatric ED.
Hripcsak **et al*. *(2007)	America	To assess how clinical information from previous visits is used in the emergency department	Accessibility	Quantitative/ Cross-sectional study	Common data types were used up to 5% to 20% from the ED, but not a majority of the time. Less than half the time, even when the user was notified of the availability of data, other data were used.
Hunt **et al*. *(2007)	America	To evaluate the completeness and accuracy of E codes for work-related and non-work-related injuries reported to a statewide Emergency Department Injury Surveillance System (EDISS)	Completeness, Accuracy	Quantitative/ Cross-sectional study	E-codes reliably identified the mechanism of injury, but their inaccuracies and incompleteness suggested areas for training of hospital admissions staff, providers, and coders
Gorelick **et al*. *(2007)	America	To determine the agreement on final diagnoses between two sources, electronic administrative sources and manually abstracted medical records, for pediatric ED visits in a multicenter network	Agreement	Quantitative/ Cross-sectional study	Overall, 67% of diagnoses from the administrative and abstracted sources were within the same diagnosis group. Agreement varied by site, ranging from 54% to 77% and by diagnosis.
Brice **et al*. *(2008)	America	To determine the accuracy of EMS information in patients who activated,EMS for chest pain and to describe the types of errors committed	Accuracy, Agreement	Quantitative/ Retrospective, consecutive case series study	The use of EMS-generated demographic data demonstrates moderate agreement and linkage with hospital records. Name and date of birth are more reliable data elements for matching than social security number..
Cwinn **et al*. *(2009)	Canada	To determine the frequency and type of clinically important information gaps for patients transferred to an emergency department (ED) from a nursing home or senior's residence. To determine the impact of a regional transfer form on the rate of information gaps	Completeness	Quantitative/ Cross-sectional study	When the standardized transfer form was used, information gaps were seen in 74.9% of transfers compared with 93.5% of the transfers when the form was not used (p < 0.001).
Mears **et al*. *(2010)	America	To create and validate a linkage of the North Carolina EMS Data System (NC-EMS-DS) with data contained in the North Carolina Stroke Care Collaborative (NCSCC) Registry	Agreement, accuracy	Quantitative/ Cross-sectional study	Matching between (NCSCC) Registry with the North Carolina EMS Data System (NC-EMS-DS) was (63%). Most verification failures were due to incorrect date ⁄ time stamp and inability to find a corresponding EMS record.
Porter **et al*. *(2010)	America	To determine if a patient-driven health information technology called ParentLink produced higher-quality data than documentation completed by nurses and physicians	Completeness Accuracy Validity	Quantitative /quasi-experimental interventional study	Parents’ valid reports of allergies to medications were higher than those of nurses and physicians. ParentLink produced more complete information on History Patient Illness(HPI) for head trauma than the medical records.
Koronios **et al*. *(2010)	Australia	To discuss the actual data quality issues with the operation-level and middle-level managers emerged during the ED dashboard development projects.	Accuracy Timeliness Consistency Completeness Integrity Conformity	Qualitative/ Literature review	Data quality issues were summarized under the well-known technology, organization, people (TOP) model, that provided guidance on the types of data that needed to be collected and required quality dimensions for reliable decision-making.
Xie **et al*. *(2010)	America	To define dimensions for describing information quality deficiencies concerning the information flow across units from the communication center to dispatch center, to mobile rescue units, and to emergency department (ED)	Timeliness, Completeness, Accuracy, Conciseness Relevancy, Accessibility Understandability Privacy, Security	Qualitative/ Literature review	A list of eight dimensions were defined from literature and used in describing information quality deficiencies in EMS performance of three cases.
Dalawari **et al*. *(2011)	America	To determine whether the use of a transfer from increases the availability of essential information needed for patient care and to examine its effect on case resolution time and disposition status	Completeness	Quantitative/ Retrospective review	Essential information for providing emergency department patient care was significantly increased with the use of a transfer form.
AbuYassin **et al*. *(2011)	Saudi Arabia	To investigate the role of pharmacists in identifying discrepancies in medication histories at admission to a tertiary referral hospital in Saudi Arabia.	Completeness, Accuracy	Qualitative/ Prospective Observational study	The most common omissions were related to medications (35%) and dosage errors (35%). Pharmacists could potentially play a major role in obtaining medication history at the time of hospital admission.
Remen and Grimsmo (2011)	Norway	To study information access and information needs in inpatient emergency departments, and how clinicians in these departments handle deficits in available information.	Completeness	Quantitative/Observational study	Information medications and past medical history were described in most referrals. For a significant number of patients the examining doctor believed that information gaps had clinical implications.
Liaw **et al*. *(2012)	Australia	To estimate the reliability of “principal diagnosis” to identify people with diabetes mellitus (DM), cardiovascular diseases (CVD), and asthma or chronic obstructive pulmonary disease (COPD) in Firstnet, the emergency department (ED) module of the Electronic Medical Record (EMR) in NSW health	Accuracy, Concordance	Qualitative/ Literature review	The incomplete concordance of diagnoses of the selected chronic diseases generated *via *different modules of the same information system raises doubts about the reliability of data and information quality collected, stored and used by the EMR.
Gao **et al*. *(2012)	Australia	To adopt a process-oriented approach to understand how data quality issues emerged through the ED data collection and reporting processes.	Completeness, Consistency Timeliness Accuracy Integrity Conformity	Qualitative/ Literature review	The development of the ED process maps is central to a comprehensive data quality assessment. These process maps will not only serve as a roadmap of where to look for data quality problems, but would also allow for possible optimization of information resources
Ward **et al*. *(2013)	America	To assess operational data quality in an emergency department (ED) immediately before and after an EHR implementation	Accuracy Timeliness	Quantitative/ Cross-sectional study	Using electronic timestamps for operational assessment and decision making following implementation should recognize the magnitude and compounding of errors when computing service times.
Hu e*t al. *(2014)	America	To test the hypothesis that the analysis of continuous vital signs acquired automatically, without prehospital provider input, improves vital signs data quality, and changes Trauma Injury Severity Scores compared with retrospectively compiled prehospital trauma registry data.	Accuracy	Quantitative/ Cross-sectional study	Continuous vital signs acquisition (VDSR technology) captures more extreme perturbations than trauma registry. The use of this technology may also lead to the development of better trauma prognostic models.
Murphy**et al*. *(2014)	America	Understanding the cause of information problems and the impact that they can have on the hospital’s workflow in ED.	Accuracy, Timeliness, Consistency, Completeness, Availability	Qualitative/ Observation and interview study	Information problems impact the collaborative patient-care including the cascading workflow effects and ambiguous accountability.
Morphet**et al*, *(**2014)	America	To investigate the documentation of resident transfers to ED, and the effect transfer documentation on the resident ED journey	Completeness	Quantitative/ Retrospective review study	The reason for transfer to the ED (48.2%); baseline cognitive function (59.7%); and vital signs at time of complaint (69.9%) were missing.
Sundermann**et al*. *(2015)	Australia	To evaluate the accuracy of PCR in the detection two critical resuscitation events, ROSC and RA, and to compare it with the capabilities of ECG and other signals recorded on the defibrillator monitor	Accuracy	Quantitative/ Cross-sectional study	PCRs were insufficient in capturing ROSC and RA events. Inaccuracy in reporting the post-RA ECG rhythm reflects the lack of texture that may be present in PCR data as well.
Dawson **et al*. *(2015)	Australia	To determine whether it is possible to collect episode-level data at six small rural emergency services (EDs) and quantify the accuracy of eight fields.	Completeness, accuracy	Quantitative/ Prospective cross-sectional study	Data entry accuracy was high for all fields audited, and data entry completeness was low for procedures.
Coffey **et al*. *(2015)	America	To compare the completeness of paper documentation with that of electronic documentation for trauma resuscitations	Completeness	Quantitative/ Cross-sectional study	Electronic documentation produced superior records of pediatric trauma resuscitations compared with paper documentation
Ward **et al*. *(2015)	America	To estimate how data errors in electronic health records (EHRs) can affect the accuracy of common emergency department (ED) operational performance metrics	Completeness, Accuracy, Timeliness	Quantitative/ Cross-sectional study	Infrequent and small-magnitude data errors in EHR time stamps can compromise a clinical organization's ability to determine it accurately.

**Table 2:** - Definition of dimensions of data quality.

**Dimension**		**Definition**
Accuracy		The extent to which data is correct and reliable. (1, 2)
Completeness		The extent to which data is not missing and in of sufficient breadth and depth for the task at hand. (1, 2)
Timeliness		The extent to which the data is sufficiently up to data for the task at hand. (1, 2)
Accessibility		The extent to which data is available, or easily and quickly retrievable. (1, 2)
Consistency		Representation of data values remains the same in multiple data items in multiple locations. (1, 2)
Errors	Omission	Occasionally, correctness included completeness, due to the fact that some researchers consider missing data to be incorrect (ie, errors of omission)/missing data. (3)
	Commission	Discrepancy or inaccurate (3)
Inaccurate/error		Error terms that were commonly used to describe accuracy and quality.(3)

**Table 3:** Data quality dimensions and their related characteristics in the reviewed papers.

Article	Characteristic		Dimension
Dawson *et al*. (26), Nagurney *et al*. (30), Hunt *et al*., (37), Kanegaye *et al*. (50), Morphet *et al*. (38), Considine *et al* . (34), Smith *et al*. (42), Dalawari *et al*. (51)	Completeness		Completeness (16 articles)
Porter *et al*. (7), Ward *et al*. (28); Abuyassin *et al*. (54), Ward *et al*. (48)	Missing data/omission		
Stiell *et al*. (39), Cwinn *et al*. (35), Remen and Grimsmo (47)	Information gaps		
Porter *et al*. (7)	Validity (accurate and complete )		Accuracy (13 articles)
Porter *et al*. (46)	False positive and negative,		
	True positive		
Brice *et al*. (33), Hunt *et al*. (37), Hu *et al*. (52), Liaw *et al*. (59)	Exact matching /agreement		
Porter *et al*. (7)	Over-reporting , under-reporting		
Ward *et al*. (28)	Accuracy		
Abuyassin *et al*. (54)	Commission		
Dawson *et al*. (26), Ward *et al*. (48)	Inaccurate/error		
Hu *et al*. (52)	Reliability		
Nagurney *et al*. (30), Sunderman *et al*. (43), Jones *et al*. (32), Porter *et al*. (46)	Sensitivity		
Nagurney *et al*. (30), Jones *et al*. (32), Porter *et al*. (46)	Specificity		
Jones *et al*. (32)	Added value		
Aronsky and Haug (25), Mckenzie *et al*. (27), Liaw *et al*. (59)	Concordance		Consistency (9 articles)
Downing *et al*. (40), Gorelick *et al*., (36), Mears *et al*. (41), Brice *et al*. (33), Hu *et al*. (52)	Linkage data	Agreement	
Hunt *et al*. (37)	Exact matching		
Aronsky and Haug (25), Gorelick *et al*. (45), Ward *et al*. (48)	Availability		Accessibility (4 articles)
Hripcsak *et al*. (57)	Accessibility		
Ward *et al*. (28), Ward *et al*. (48), Travers *et al*. (49)	Timeliness		Timeliness (3 articles)
